# Text-Book of Therapeutics and Materia Medica

**Published:** 1889-04

**Authors:** 


TEXT BOOK OF THERAPEUTICS AND MATERIA MEDICA, INTENDED FOR THE USE OF STUDENTS AND PRACTITIONERS. By Robert T. Edes, A. B., M. D. Philadelphia: Lea Bros. & Co. 1888, pp. 552.

